# Dynamic control of the plasmid copy number maintained without antibiotics in *Escherichia coli*

**DOI:** 10.1186/s13036-024-00460-1

**Published:** 2024-12-19

**Authors:** Geunyung Park, Jina Yang, Sang Woo Seo

**Affiliations:** 1 Interdisciplinary Program in Bioengineering, Seoul National University, 1 Gwanak-ro, Gwanak-gu, Seoul, 08826 Republic of Korea; 2 Department of Chemical Engineering, Jeju National University, 102, Jejudaehak-ro, Jeju-si, Jeju-do 63243 Korea; 3 School of Chemical and Biological Engineering, Seoul National University, 1 Gwanak-ro, Gwanak-gu, Seoul, 08826 Republic of Korea; 4 Institute of Chemical Processes, Seoul National University, 1 Gwanak-ro, Gwanak-gu, Seoul, 08826 Republic of Korea; 5 Bio-MAX Institute, Seoul National University, 1 Gwanak-ro, Gwanak-gu, Seoul, 08826 Republic of Korea; 6 Institute of Bio Engineering, Seoul National University, 1 Gwanak-ro, Gwanak-gu, Seoul, 08826 Republic of Korea

**Keywords:** Plasmid copy number, Dynamic control, Essential gene, Antibiotics-free, Metabolic engineering, Gene regulation

## Abstract

**Background:**

Manipulating the gene expression is the key strategy to optimize the metabolic flux. Not only transcription, translation, and post-translation level control, but also the dynamic plasmid copy number (PCN) control has been studied. The dynamic PCN control systems that have been developed to date are based on the understanding of origin replication mechanisms, which limits their application to specific origins of replication and requires the use of antibiotics for plasmid maintenance. In this study, we developed a dynamic PCN control system for *Escherichia coli* that is maintained without antibiotics. This is achieved by regulating the transcription level of the translation initiation factor IF-1 (*infA*), an essential gene encoded on the plasmid, while deleting it from the plasmid-bearing host cell.

**Results:**

When validated using GFP as a reporter protein, our system demonstrated a 22-fold dynamic range in PCN within the CloDF13 origin. The system was employed to determine the optimal copy number of the plasmid carrying the *cad* gene, which converts an intermediate of the tricarboxylic acid cycle (TCA cycle) to itaconic acid. By optimizing the PCN, we could achieve an itaconic acid titer of 3 g/L, which is 5.3-fold higher than the control strain.

**Conclusions:**

Our system offers a strategy to identify the optimal expression level of genes that have a competitive relationship with metabolic pathways crucial for the growth of the host organism. This approach can potentially be applied to other bacterial hosts by substituting the sensing module or the essential gene.

**Supplementary Information:**

The online version contains supplementary material available at 10.1186/s13036-024-00460-1.

## Background

Overexpressing the genes in the synthesis pathway is a common strategy to obtain a high titer of a target metabolite. However, each metabolite producing gene has its optimal expression level for the best production. Overexpression of genes does not always guarantee the expected result. For example, the overexpression of genes could lead to growth inhibition or toxicity in the host bacteria [1, 2]. Therefore, the optimization of gene expression levels in metabolic engineering is important, and this has been conducted at transcriptional, translational, or even post-translational levels. At the transcriptional level, gene expression can be modulated by replacing a promoter with a static promoter or inducible promoter or by using the CRISPRi system [3–8]. To modulate protein translation efficiency, ribosome binding sites with appropriate strength can be designed or selected from a library [9–11]. Alternatively, translation can be down regulated using antisense RNA [12], small RNA [13, 14], or Tl-CRISPRi [15]. The amount of protein can also be modulated at the post-translational level. Degradation tags such as ssrA and Pup, or degrons have been used in conjunction with their partner proteases to enhance the degradation of target proteins [16–18].

Recent studies have shown that by altering the components of the plasmid origin of replication, gene dosage can be regulated through dynamic control of plasmid copy number (PCN) [19–21]. This approach reduces the requirement for multiple cloning steps to determine the optimal expression levels of the genes residing in the same plasmid. By substituting the promoter of the plasmid replication initiation factor with one regulated by cuminic acid to regulate the inhibitory transcription factor of the promoter, cuminic acid-dependent PCN change was enabled [19]. In the case of pUC19 origin, the PCN was manipulated by tuning the priming and inhibitory RNA working on the replication origin, and the system was applied to the violacein production [20]. In addition, the PCN within ColE1 and p15A origin could be regulated in two plasmids system in which one plasmid regulated the transcription of either priming or inhibitory RNA of the origin on the other plasmid [21]. However, all the aforementioned static or dynamic gene expression systems still necessitate the use of antibiotics for plasmid maintenance.

The use of antibiotics in metabolite production has several disadvantages. Plasmid maintenance with antibiotics can lead to the emergence of antibiotic-resistant organisms [22] and cause PCN heterogeneity [23]. Especially when producing compounds intended for human contact or ingestion, reducing antibiotics during the production process is preferable to ensure safety. Previous studies demonstrated the stable maintenance of plasmids without antibiotics by applying toxin-antitoxin mechanism [24, 25], auxotrophic or essential gene complementation [26, 27], or repressor titration [28]. Especially, STAPL system that maintains plasmids within cells without using antibiotics by relocating essential gene, translation initiation factor IF-1 (*infA*), to the plasmid and removing the gene from the chromosomes was developed [29]. The study also discovered that the PCN shifts in response to static changes in *infA* transcription levels [29]. The PCN and expression levels of the essential gene exhibited an inverse correlation.

In this study, we developed a dynamic PCN controlling system maintained without antibiotics by dynamically modulating the transcription level of a plasmid-relocated essential gene, *infA*. The transcription level of *infA* is controlled by an inverting genetic circuit in which anhydrotetracycline (aTc) addition leads to the reduction in *infA* expression level. The system was designed to maintain maximum *infA* expression during the cloning step, keeping PCN low. For the production of target metabolite, PCN can be upregulated by adding an inducer to reduce *infA* expression. The developed system was applied to the optimization of itaconic acid production.

## Methods

### Strains, plasmids, primers, genetic manipulation

Mach-T1^R^ was used for the construction of plasmids used in this study. Otherwise, MG1655 was used. The strains and plasmids used in the study are listed in Table S1. The primers used for the construction of plasmids are listed in Table S2. Gene parts for *tetR* (TetR-HpaI-R, phlF-TetR-R), *phlF* (phlF-TetR-F, phlF-R1) (phlF-TetR-F, phlF-R2), *gfp* (opt-gfp-R, opt-gfp-F) (opt-gfp-R, P_*phlF*_-phlF-F), *infA*(InfA- P_*phlF*_ -F, InfA-PstI-R) (InfA-PstI-R, P_*phlF*_ -phlF-F), were amplified once or twice using PCR and assembled using NEBuilder HiFi DNA Assembly kit (New England Biolabs, Ipswich, MA, USA) with Bsu36I, HpaI cut pCDF-Duet vector. This vector was used as a template and modified accordingly for other plasmids used in this study. For the tight regulation of the dynamic control genetic circuit, BBa_B1006 (90% efficiency) was chosen as the terminator for *phlF*. In addition, the RBS of *phlF* was set to the expression level around 40,000 using the UTR designer [10].

In order to knock out the *infA* gene on the chromosome, a plasmid containing *infA* and the induction module (*tetR* and *phlF* with *tet* promoter, *infA* with *phlF* promoter) was electroporated to MG1655_pSIM5. The editing template containing the kanamycin resistance gene and FRT sequences was prepared by PCR with primers (D-infA-F2, D-infA-B2) and electroporated to the lambda red competent cell. The cells undergone successful recombination were selected on LB plates containing spectinomycin and kanamycin.

### Specific fluorescence measurement

Modified M9 medium (47.8 mM Na_2_HPO_4_, 22.0 mM KH_2_PO_4_, 18.7 mM NH_4_Cl, 8.6 mM NaCl, 2 mM MgSO_4_, and 0.1 mM CaCl_2_) containing 4 g/L glucose and 7.5 g/L casamino acids was used.

The regulation range of *phlF* promoter in *E. coli* DCP strain (Supplementary Table. S1) was measured with Sense (Hidex, Turku, Finland). Overnight incubation was done in 5 mL medium in 100 mL test tube at 37 °C, 250 rpm. The incubated cells were diluted to OD_600_ 0.1 and grown until OD_600_ exceeds 1.0. The cells were inoculated to OD_600_ 0.05. When OD_600_ reached around 0.3, aTc was added to the final concentration of 0.01, 0.1, 1, 2. 3, 5, 10, 50, 100, 500, and 1000 ng/mL. The specific fluorescence was measured by the dividing fluorescence value by the measured OD_600_ at 10 h after aTc addition.

The GFP measurement in our dynamic control of the PCN system was conducted in *E. coli* DCG strain (Supplementary Table. S1) using S3e Cell Sorter (Bio-Rad, California, USA). Overnight inoculation was prepared in 5 mL medium in a 100 mL test tube. The incubated cells were refreshed to OD_600_ 0.1, grown to exponential phase, and inoculated to OD_600_ 0.05. When OD_600_ reached around 0.3, aTc was added to the final concentrations of 0.05, 0.5, 5, 50 ng/mL in 5 mL. After 10 h of incubation from the aTc addition, the specific fluorescence was directly measured using flow cytometry.

### Quantification of the PCN

The copy number was quantified using quantitative PCR (qPCR) following a previously used method and modified accordingly [30]. Accupower 2X greenstar qPCR Master Mix (Bioneer, Daejon, Republic of Korea) was used for the reaction mixture. StepOnePlus Real-time PCR system (Thermo Fisher Scientific, Waltham, MA, USA) was used.

For the standard curve development, MG1655 gDNA and pCDF-Duet vector were prepared. The *rpoA* flanking region was amplified with Flank_rpoA_eco_F and Flank_rpoA_eco_R, and was used as the gDNA template. The concentrations of pCDF-Duet vector and *rpoA* flanking region of gDNA were measured using Qubit assay. The primers qPCR_cloDF13_F, qPCR_cloDF13_R and qPCR_rpoA_eco_F, qPCR_rpoA_eco_R were used respectively for serially diluted pCDF-Duet and *rpoA* flanking region (Supplementary Fig. S4).

Cell cultures diluted in DDW were boiled at 95 °C for 10 min and diluted to OD_600_ 0.01. Each sample was amplified using pCDF-Duet vector primers and gDNA primers. PCN was calculated by dividing the plasmid molecules by gDNA molecules.

### Itaconic acid production

The strain used for the production of itaconic acid (DCI (Supplementary Table. S1)) was grown in an M9 medium supplemented with 2 g/L yeast extract, 10 mL/L ATCC trace mineral solution, and 20 g/L glucose at 30 °C, 200 rpm. Kanamycin (50 $\:\mu\:$g/mL) was used to prevent contamination. Each seed was inoculated in a 5 mL medium contained in a 100 mL test tube. Overnight cultured cells were diluted to OD_600_ 0.1 in a 10 mL medium contained in a 300 mL flask. The cells were inoculated to OD_600_ 0.05 in 25 mL of medium in a 300 mL flask, and aTc (final concentration: 0.05, 0.5, 5, 50, 500 ng/mL) was added when OD_600_ reached approximately 0.3. The pH titration was conducted every 6 h after the aTc addition, to approximately pH 7.0. All samples were prepared in biological triplicates.

### HPLC analysis

For the quantitative analysis of itaconic acid, the supernatant of the cell culture was filtered and analyzed using HPLC (Shimadzu, Kyoto, Japan) with cation-exchange Aminex HPX-87 H column (Biorad, California, USA) [31]. 5 mM sulfuric acid was used as the mobile phase. The flow rate was 0.6 mL/min and the oven temperature was maintained at 14 °C. Glucose was detected with a refractive index detector, and acetic acid and itaconic acid were detected with a UV–vis diode array detector (at 210 nm). For HPLC standards, itaconic acid was purchased from TCI America, glucose was purchased from Acros Organics, and acetic acid was purchased from Supelco.

## Results

### Design of dynamic PCN control system

**Fig. 1:**
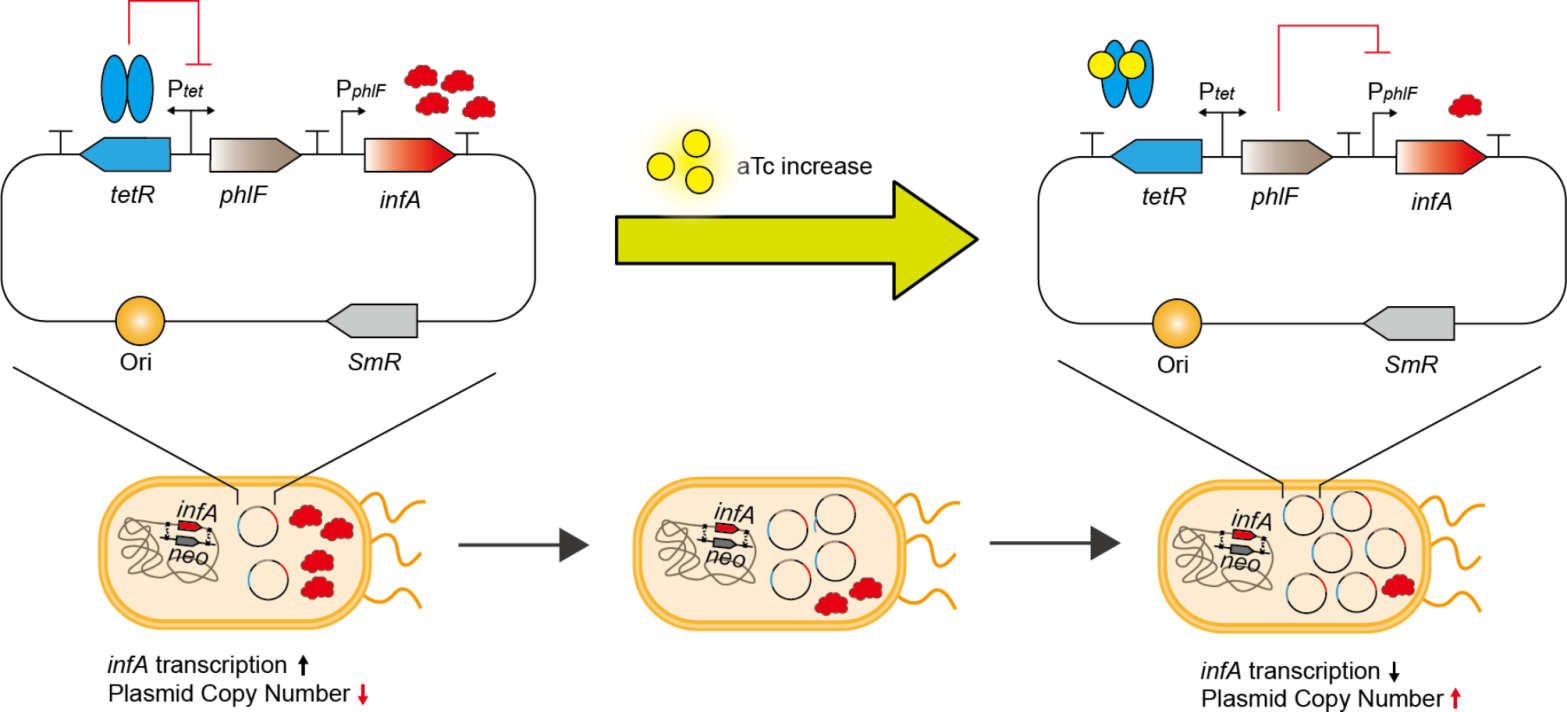
The overall scheme of the dynamic control of PCN. Without aTc, TetR binds to the operator region of *phlF* promoter, repressing *phlF* expression. Therefore, the comparably enough *infA* expression will lead to less PCN. When aTc is added, aTc binds to TetR, unrepressing *phlF* expression. Expressed *phlF* then represses the *infA* expression, upregulating the PCN

A genetic circuit which enables the dynamic PCN control within an origin of replication was devised (Fig. 1). The previous study discovered that the static change in the transcription level of *infA* led to the alteration in the PCN [29]. According to the study, the PCN was inversely proportional to the transcription level of *infA*. For metabolite production, the expression level of the corresponding gene is usually maintained low during the cloning process and is induced during the production stage to minimize the burden posed on the cell. Therefore, we designed a system that keeps PCN low without an inducer and increases PCN at specific time points with the addition of an inducer. We employed an inverted induction module by which the inducer addition lowers the *infA* expression level, thereby upregulating PCN. Therefore, we combined two activating inducible systems to reverse the signal of the induction and repress *infA* expression. For future applications, it is sufficient to change only the primary sensing module while keeping the *infA* promoter unchanged. This approach will relieve us of the need for repeated optimization and validation of the *infA* promoter each time.

We selected a regulatory system consisting of a PhlF promoter (P_*phlF*_) and tetA promoter (P_*tetA*_), in which the PhlF repressor is controlled by the P_*tetA*_ and repressed by the TetR (Fig. 1). The addition of aTc activates the expression of *phlF*. Subsequently, P_*phlF*_ driven *infA* expression is repressed [32]. There is a strong correlation between *infA* and cell growth; not only is the transcription of *infA* associated with cell growth [33, 34], but *infA* is also essential for cell viability [35]. As a result, a decrease in *infA* expression leads to an increase in PCN to maintain the necessary levels of *infA*. Since *infA* is a critical gene, insufficient expression hampers cell growth, causing an accumulation of plasmids within the cell. Conversely, since *infA* expression is not repressed in the absence of aTc, cells are expected to retain enough amount of translation initiation factor to survive without increasing the PCN (Fig. 1).

## Validation of the designed dynamic PCN control system

**Fig. 2:**
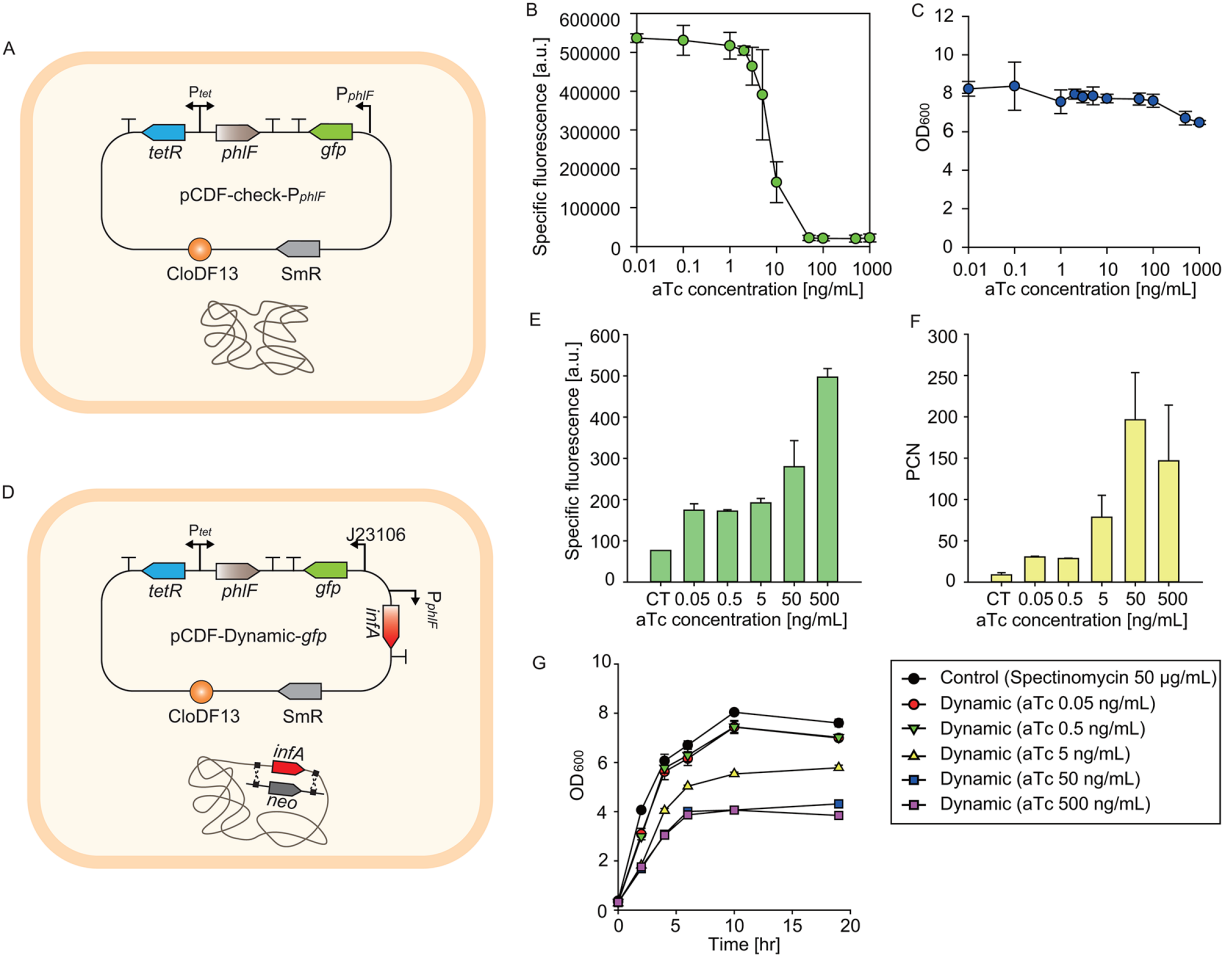
Validation of our dynamic control of PCN system. (**A**) Design of P_*phlF*_ dynamic range check plasmid construct in DCP. (**B**) GFP specific fluorescence regulation range of the *phlF* promoter at 10 h after aTc (0.01, 0.1, 1, 2, 3, 5, 10, 50, 100, 500, 1000 ng/mL) addition. (**C**) Cell growth effect (OD_600_) of DCP at 10 h after aTc induction. (**D**) Dynamic control of the PCN cassette design in DCG. (**E**) Mode of GFP specific fluorescence measured by flow cytometry at 10 h after aTc (0.05, 0.5, 5, 50, 500 ng/mL) addition. Spectinomycin was only used in the control strain. (**F**) Biological triplicate results of PCN at 10 h measured by qPCR. (**G**) Cell growth (OD_600_) of the control and DCP with aTc (0.05, 0.5, 5, 50, 500 ng/mL)

The inverted induction module was first tested in DCP. The regulation range of P_*phlF*_ by the addition of aTc was tested by placing *gfp* under the control of the promoter (Fig. 2A). The specific fluorescence was measured to deduce the regulation range of the inverted induction module. As the concentration of aTc increased, the specific fluorescence exhibited a marked decline beginning at 50 ng/mL, showing a 24-fold reduction. At higher concentrations of aTc, the specific fluorescence remained consistent, with the maximum reduction reaching 26-fold (Fig. 2B). However, cell growth was inhibited above 500 ng/mL aTc, which is known to have cytotoxicity [36] (Fig. 2C). To avoid reducing cell growth by more than 25%, aTc concentrations below 500 ng/mL were used in the following experiments.

Next, we examined how the PCN changed in response to the *infA* expression level (Fig. 2D). The PCN of DCG was indirectly measured by the constitutively expressed *gfp*. The specific fluorescence was measured by flow cytometry as it segregates each cell into individual droplets and yields a precise fluorescence each cell possesses [37]. The control strain (SCG) was cultured in spectinomycin containing condition, as we aimed to compare our system to the conventional plasmid maintaining system. The specific fluorescence elevated as the concentration of aTc increased, which indirectly indicated the increase in the PCN. The DCG strain showed a 6.5-fold regulation range of specific fluorescence compared to the control strain, when aTc 500 ng/mL was added (Fig. 2E, Supplementary Fig. S1). The qPCR result demonstrated the actual PCN had a 22-fold regulation range compared to the control strain when aTc 50 ng/mL was added (Fig. 2F). The regulation range of PCN (9-196 copies, 22-fold) is much broader compared to the reported copy number range of CloDF13 (20–40 copies, 2-fold) [38]. Therefore, it was confirmed that adjusting the expression level of *infA* can dynamically regulate PCN, thereby enabling control over the expression level of the target protein.

The dynamic range of the specific fluorescence was less than that of the PCN. A plausible reason for this phenomenon would be the role of *infA*, which is a translation initiation factor. Too low *infA* expression due to the addition of a high concentration of aTc might impair the efficiency of translation initiation in the cell. This could be improved in further studies to encompass a wider range of target protein expression levels. Although the specific fluorescence was highest at 500 ng/mL of aTc, the PCN was lower than that at 50 ng/mL of aTc. P_*phlF*_ was effectively turned off at aTc 50 ng/mL (Fig. 2B). The deviation from the expected increase in PCN at aTc 500 ng/mL may have resulted because this concentration exceeded the regulatory range. One hypothesis is that the repressor of primer (Rop) protein, which is encoded on a dynamically regulated plasmid, accumulates beyond a certain level like GFP. This may enhance the formation of kissing complexes or repress the transcription of RNAII thereby reducing the PCN. Yet, we are not certain of the exact mechanism. The major reason for the cell growth inhibition (Fig. 2G) was considered to be *infA* expression level reduction as the reduction in cell growth was two times greater than the aTc cytotoxicity effect (Fig. 2C). The real-time quantitative reverse transcription PCR (qRT-PCR) results indicated that cells maintained *infA* mRNA levels up to 5 ng/mL of aTc, but these levels decreased to 60% at 50 ng/mL (Fig. S2A), likely contributing to growth inhibition. Slight growth inhibition at 5 ng/mL aTc was due to increased PCN to restore *infA* levels. The expression level of the *infA* transcript from each plasmid (Fig. S2B) corresponded with the relative fluorescence intensity of DCP (Fig. 2B). This demonstrates that variations in *infA* expression levels affected both PCN and cell growth. Overall, the genetic circuit consisting of two inducible promoters allowed the adjustment of PCN by modulating the *infA* expression level through simple changes in inducer concentration.

### Application of the dynamic PCN control system in metabolite production

Dynamic PCN control system can be applied to determine the optimal PCN for metabolite production. The optimal expression level of enzymes is often difficult to predict, depending on the metabolic pathway. A specific expression level, which may include low expression of the target gene, often results in higher titer [2, 39, 40]. The PCN control system minimizes the need for redundant cloning typically required to adjust PCNs, thereby simplifying the process of optimizing the expression level of the target gene. This optimization is crucial when the target gene competes with the metabolic pathways related to cell growth in the host bacteria. The system can be applied not only to cell growth-related pathways but also to other metabolic pathways that are difficult to delete or engineer to reduce flux. This approach ensures the efficient balance of gene expression and metabolic activities necessary for optimal bacterial function and chemical production. Additionally, our developed system can incorporate the optimization of PCN when there is a discrepancy between PCN and protein expression levels.

**Fig. 3:**
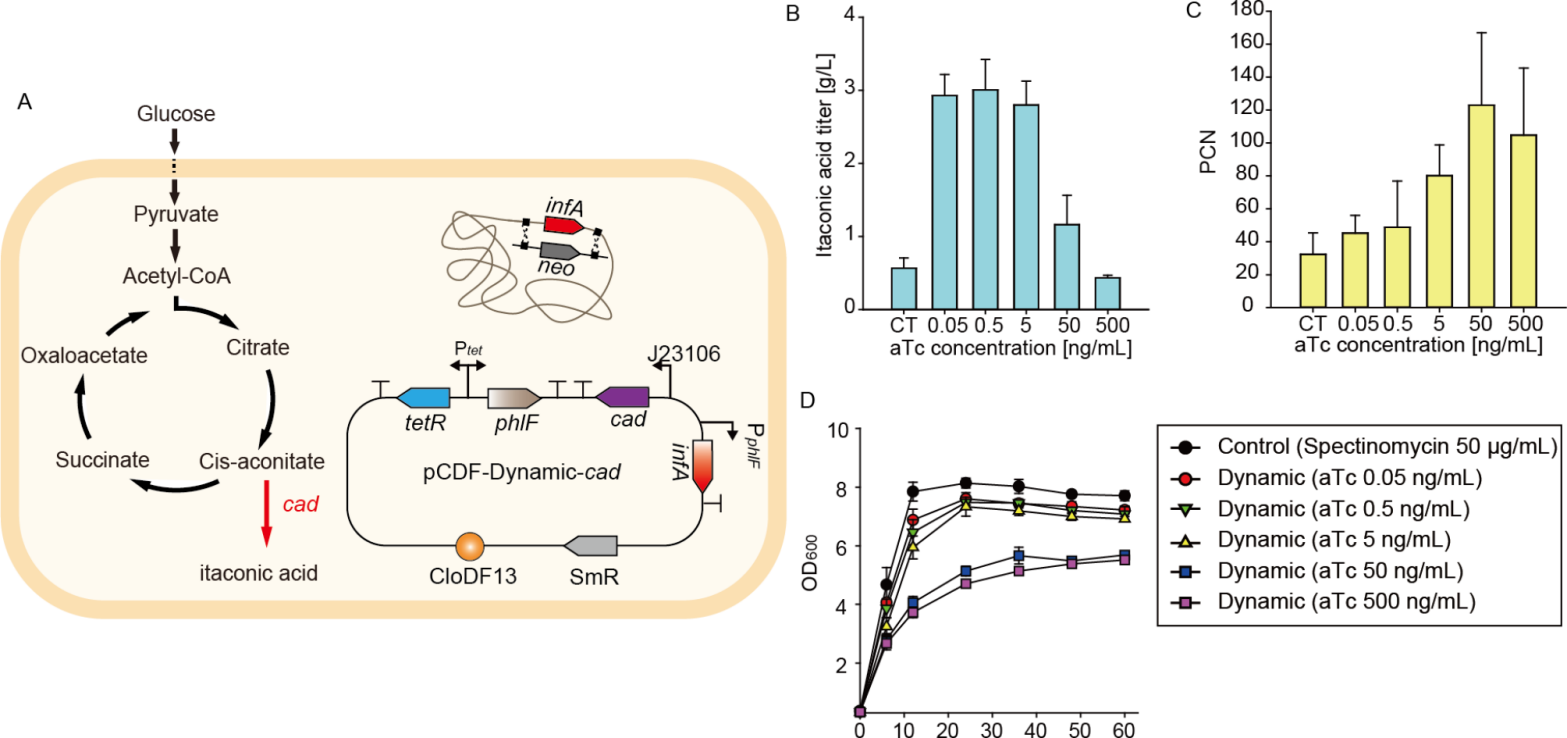
Application of our dynamic control of plasmid control system in the production of itaconic acid. (**A**) Itaconic acid production metabolic pathway in DCI. (**B**) Itaconic acid titer at 60 h when various concentrations of aTc (0.05, 0.5, 5, 50, 500 ng/mL) were added. Spectinomycin was only used in the control strain. (**C**) The PCNs in itaconic acid producing strains when aTc was added. (**D**) Cell growth (OD_600_) when aTc was added

We applied our system for itaconic acid production, a platform chemical for high-value products such as plastics and latex [41, 42]. The synthesis pathway of itaconic acid competes with the tricarboxylic acid (TCA) cycle through cis-aconitate. Cis-aconitate is a substrate for cis-aconitate decarboxylase (CAD) and is also an intermediate in the TCA cycle. Therefore, the optimal flux distribution is crucial to achieve a high titer of itaconic acid. The CAD, which enables the conversion from cis-aconitate to itaconic acid, was expressed under the control of medium strength promoter J23106 to avoid cellular burden. The gene was integrated into the plasmid, which included a dynamic PCN control cassette. Subsequently, the *infA* gene on the chromosome was deleted to yield the strain, DCI (Fig. 3A). Increase in the titers of acetate and itaconic acid was observed throughout the cultivation (Supplementary Fig. 3). The itaconic acid titer was not proportional to the pattern of PCN. However, we were able to determine the optimal copy number of DCI for maximizing itaconic acid production. The itaconic acid titer from the DCI strain was 5.3 times greater than that of the control strain (SCI). When compared to the STAPL system in the previous study, our DCI achieved 3.4 fold itaconic acid titer (Supplementary Figure. S3) [29]. This significant increase was observed when the PCN ranged from 45 to 80, with the concentration of aTc maintained between 0.05 ng/mL and 5 ng/mL (Fig. 3B and C). To analyze the expression of CAD, we performed SDS-PAGE (Supplementary Figure S5). The expression of CAD mirrored the itaconic acid titer rather than the PCN, even though other proteins on the same plasmid (Sm^R^, PhlF, and TetR) appeared to exhibit increased expression levels in accordance with the PCN (Supplementary Figure S5 and Fig. 3C). This result is consistent with previous literature demonstrating that an increase in CAD expression leads to an increased itaconic acid titer [43]. We hypothesize that CAD expression is hindered by an increase in PCN, as it is linked to cell growth and competes in the TCA cycle, resulting in lower expression levels due to cellular burden. Although we do not fully understand the underlying mechanism, enzyme expression decrease at high plasmid copy number has been reported [2]. The culture conditions such as media composition, volume, and temperature differed between the production of GFP and itaconic acid. The trends in cell growth in response to aTc addition were analyzed [44]. When comparing the PCN in Figs. 2F and 3C, the control strain and the DCI strains at aTc concentrations of 0.05 and 0.5 ng/mL showed significantly greater PCN in DCI than in DCG. In contrast, at aTc concentrations of 5, 50, and 500 ng/mL, the PCN of DCI was similar to or slightly lower than that of DCG. This indicates that *infA* repression was comparatively less stringent at aTc concentrations of 5, 50, and 500 ng/mL. Consequently, the growth curves for DCI at 5, 50, and 500 ng/mL appeared to be elevated compared to those of DCG. To sum up, the dynamic PCN control system enabled the determination of the optimal expression of *cad*, which competes with the TCA cycle. When stronger CAD promoters are utilized or when CAD is colocalized with aconitase (Acn) [45], the titer of itaconic acid is expected to increase. This occurs as CAD fully redirects the metabolic flux from the TCA cycle toward the production of itaconic acid. The system will ease the prediction of optimal gene expression in metabolite production.

## Discussion

Recently, several studies regarding the dynamic PCN control system have been developed [19–21]. These studies modulated the genetic parts involved in the mechanism of replication in the origin. In this study, we developed a dynamic PCN control system that is not restricted to a specific origin of replication. Instead, it may be universally applied by regulating PCN through the dynamic control of the transcriptional expression level of the essential gene, *infA*. The ColE1 origin of replication including CloDF13 is primarily controlled by the RNAII to RNAI ratio. RNAII initiates replication by binding to the replication start site, while RNAI inhibits this process by forming a complex with RNAII, assisted by the Rop protein. ColE1-like origins can experience runaway replication if the RNAII/RNAI ratio is disrupted [46]. We hypothesized that reduced *infA* expression may decrease Rop levels, leading to increased PCN. Conversely, an excess of Rop could enhance complex formation, repress RNAII, and reduce PCN. The study observed PCN changes with dynamic alterations in *infA* expression, indicating that while *infA* does not directly interfere with the replication mechanism of CloDF13 origin, it influences PCN. Another hypothesis is that a decrease in *infA* expression leads to an increase in PCN to maintain essential protein levels. Insufficient expression of an essential gene hinders cell growth, resulting in an accumulation of plasmids within the cell. The qRT-PCR results support our concept: reducing *infA* expression from individual promoters led cells to increase the PCN to restore intracellular *infA* mRNA levels (Supplementary Figures S2A and S2B). The previous study [29] has succeeded in stable plasmid maintenance without antibiotics in CloDF13 origin and pMB1 origin. This strategy may extend to other ColE1-like origins with similar replication mechanisms. Given that programmed *infA* reduction upregulates PCN, this system may be particularly more applicable for origins with medium or low copy numbers. As a member of the translation initiation factors, excessively reducing *infA* expression to increase PCN results in growth inhibition and reduction in translation initiation efficiency. For further studies, other essential genes, such as those encoding elongation factor P or elongation factor G, could be used to replace *infA* to improve the dynamic range of the target gene and to enable multi-plasmid usage [47, 48].

The dynamic PCN control system can be applied to efficiently presume the optimal gene expression level within a replication of origin, reducing the number of redundant cloning to tune the expression level of the genes by changing the promoter, ribosome binding site strength for each gene on the plasmid. When our system was applied, itaconic acid titer increased 3.4-fold compared to that of the previously reported STAPL strain [29]. This demonstrated efficient dynamic flux distribution at the desirable time point by dynamically adjusting the PCN. The PCN control system, when combined with inducible promoters, can maximize the variation in target protein levels within the cell. In addition, optimizing the expression of multiple genes in synthetic pathways can be challenging due to the need to select specific inducible promoters and their corresponding inducers. The system can be applied to multiple genes on the same plasmid, achieving different expression levels. A single input signal can upregulate the entire pathway while maintaining the optimized expression ratio for each gene by modulating the PCN. This system can be further applied to various fields of metabolic engineering when the inverted induction module of the genetic circuit is replaced with other sensing systems such as quorum sensing system or metabolite responsive biosensors [49, 50]. In addition, the system has the potential to be widely used in various bacteria, the essential genes of which are revealed. These demonstrate the borad applicability of the PCN dynamic control system.

## Conclusions

In this study, we have developed the dynamic PCN control system which does not require the use of antibiotics for the plasmid maintenance. Our system maintains the plasmid and modulates its copy number by dynamically controlling the transcription of the essential gene *infA* on the plasmid while removing it from the chromosome. The optimal gene expression level can be determined by adjusting the PCN within an origin of replication through varying inducer concentrations. Overall, our system provides a method to control gene expression at the gene dosage level. This system has the potential to be adapted to various conditions, as the genetic module can be easily replaced.

## Electronic supplementary material

Below is the link to the electronic supplementary material.

Supplementary Material 1

## Data Availability

No datasets were generated or analysed during the current study.

## Abbreviations

PCN: Plasmid copy number
