# Supplementary material for: Dynamic control of the plasmid copy number maintained without antibiotics in Escherichia coli
Source: J Biol Eng. 2024 Dec 19;18:71. doi: 10.1186/s13036-024-00460-1 (PMC11660809; doi:10.1186/s13036-024-00460-1)
Supplement: Supplementary file 1 — Supplementary Material 1 [file 13036_2024_460_MOESM1_ESM.docx]

**Supplementary Materials**

**
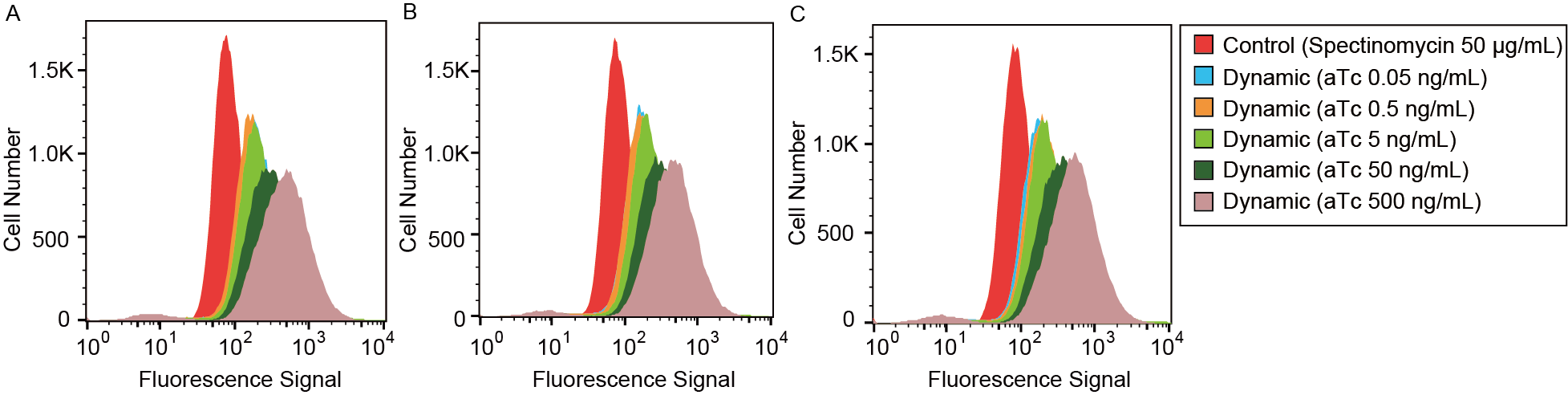
**

**Supplementary Fig. S1 Specific fluorescence measured by flow cytometry.**

Biological triplicate data of specific fluorescence in Dynamic-*gfp* strain when aTc 0.05, 0.5 5, 50, 500 ng/ml were added. The flow cytometry result was obtained when 10 hours had passed since the aTc addition. (A) The first colony (B) The second colony (C) The third colony

**
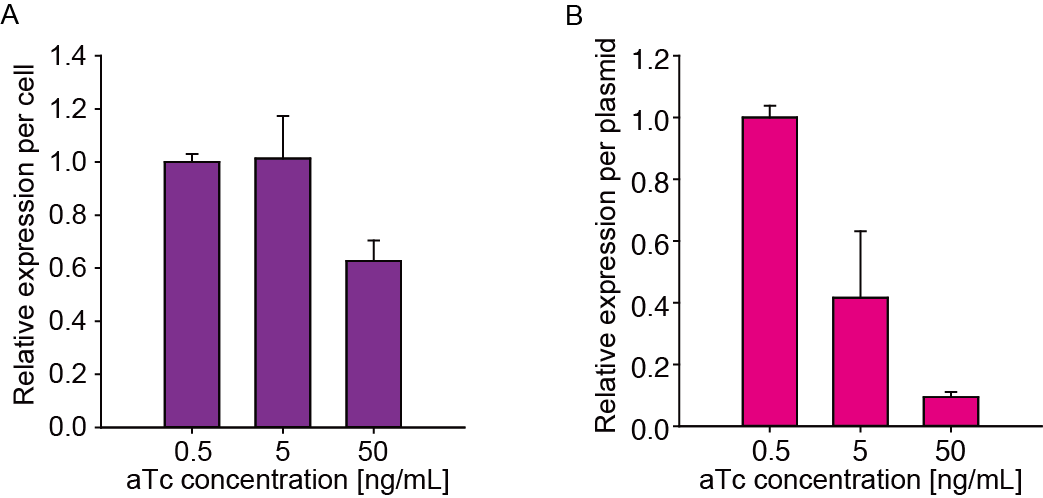
**

**Supplementary Figure S2. Change in the expression level of *infA* mRNA with increasing aTc concentration**

Total RNA was extracted from DCP culture sampled ~~at~~ 10 hours after aTc induction(0.5, 5, 50 ng/mL). After RNA extraction, the relative expression level of *infA* mRNA per plasmid was normalized to the sample induced with 0.5 ng/mL aTc. To express the amount per plasmid, the *infA* mRNA level in the cell was divided by the PCN.


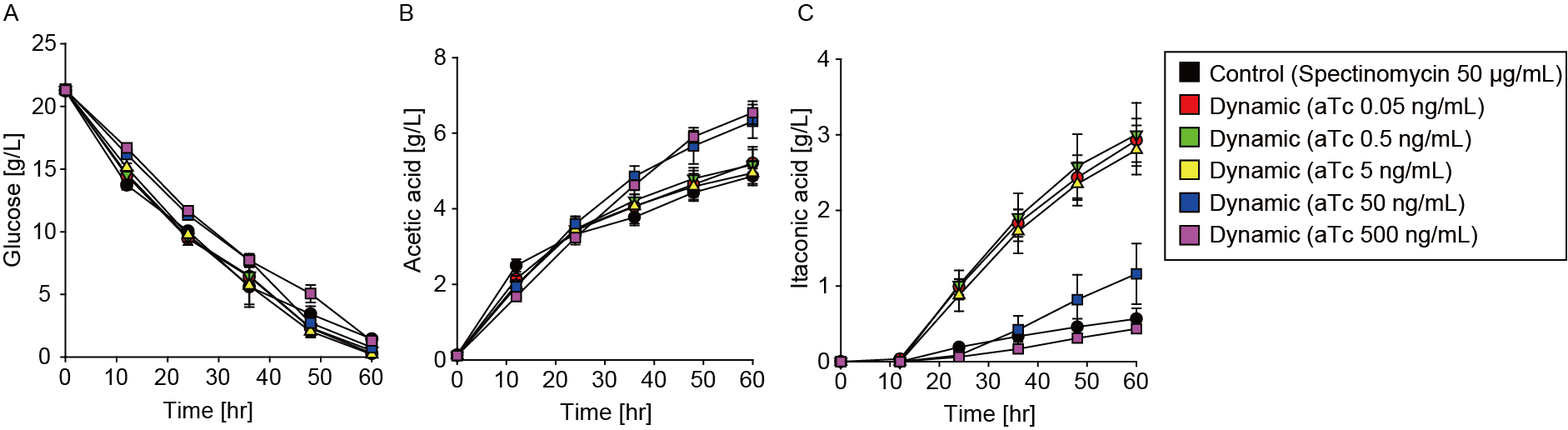


**Supplementary Figure S3. Time course glucose, acetic acid, itaconic acid titer in Dynamic-*cad* strain.**

The supernatant of culture media was sampled ~~in~~ every 12 hours. The time course titer of the sampled supernatant was filtered and analyzed in HPLC. (A) Glucose (B) Acetic acid (C) Itaconic acid

**
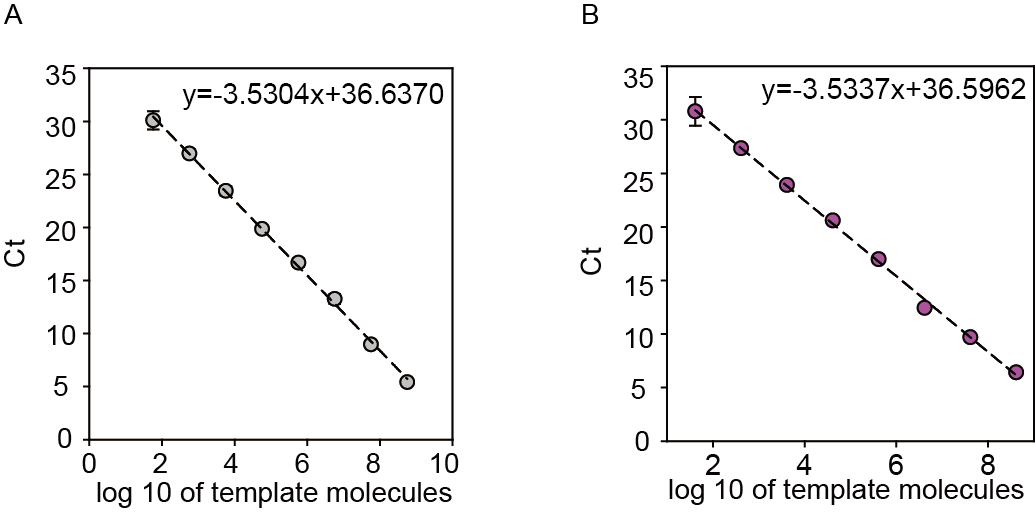
**

**Supplementary Figure S4. qPCR standard curves**

The qPCR standard curves of the chromosome and the plasmid were obtained. (A) gDNA standard curve (B) pCDF-Duet standard curve


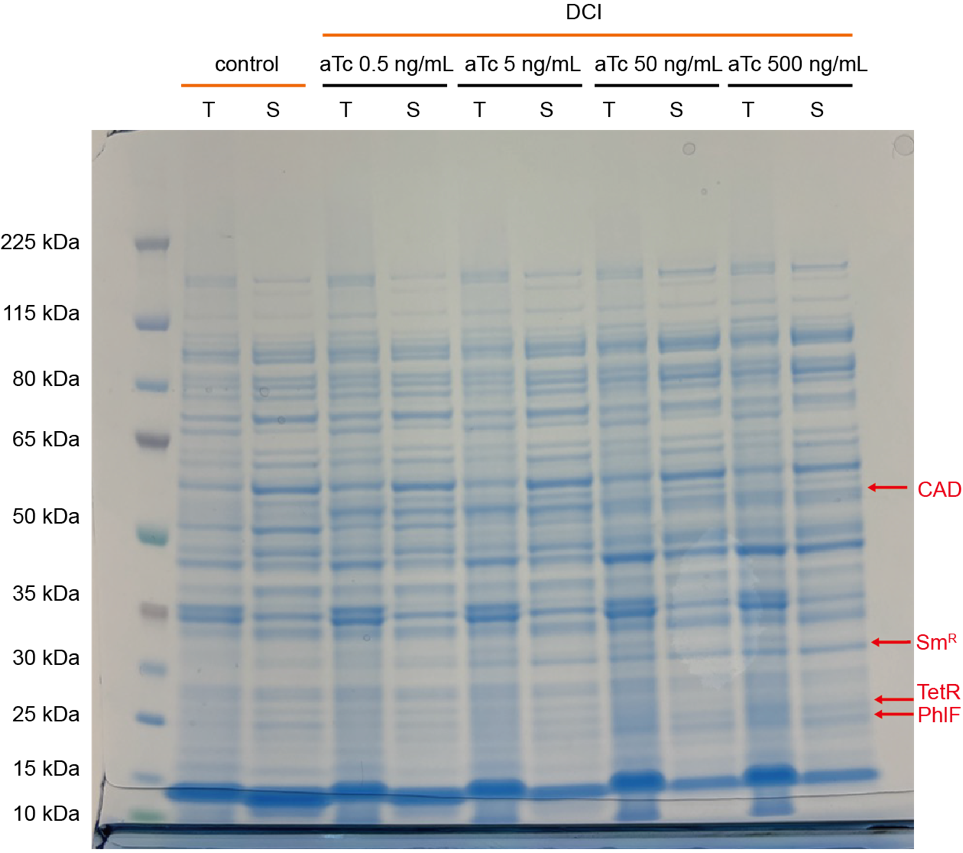


**Supplementary Figure S5. SDS-PAGE of itaconic acid producing strains**

The total and soluble fractions of 36 hour samples from the control strain and DCI strains at various aTc concentrations (0.5, 5, 50, and 500 ng/mL) were analyzed using SDS-PAGE. The target protein, Cad, is 54 kDa.

Table S1. Strains and plasmids used in this study

| Name | Relevant characteristics | Source | |
| --- | --- | --- | --- |
| **Strains** |  |  | |
| Mach-T1^R^ | *E. coli* F^-^ $\varphi$80(*lac*Z)ΔM15 Δ*lac*X74 *hsd*R(r_K_^-^m_K_^+^) Δ*rec*A1398 *end*A1 *ton*A | Invitrogen | |
| MG1655 | E. coli K-12 substr. MG1655 | ATCC 700926 |  |
| MG1655_pSIM5 | MG1655 / pSIM5 | This study | |
| MG1655_pSIM5-*gfp* | MG1655_pSIM5 / pCDF-Dynamic-*gfp* | This study | |
| MG1655_pSIM5-*cad* | MG1655_pSIM5 / pCDF-Dynamic-*cad* | This study | |
| DCP | MG1655 / pCDF-check-P*_phlF_* | This study | |
| SCG | MG1655/pCDF-*gfp* | This study | |
| DCG | MG1655 $\Delta$*infA*::FRT-*Kan^R^-FRT* / pCDF-Dynamic-*gfp* | This study | |
| DCI | MG1655 $\Delta$*infA*::FRT-*Kan^R^-FRT* / pCDF- *Dynamic*_*cad* | This study | |
| SCI  **Plasmids** | MG1655/ pCDF-*cad* | This study | |
| pCDF-Duet | Expression vector, Sm^R^, CloDF13 ori | Novagen | |
| pSIM5 | Heat-inducible lambda-red recombinase expression vector, Cm^R^ | (Datta et al, 2006) | |
| pFRT72variant | T-vector containing Kan^R^ | (Lim et al., 2013) | |
| pCDF-tetR-phlF-infA-J23106_gfp | Plasmid containing tetR/tetA promoter, phlF, gfp | This study | |
| pCDF-check-P*_phlF_* | pCDF-*tetR*-*phlF*-P*_phlF_*_*gfp* | This study | |
| pCDF-*gfp* | pCDF-*tetR*-*phlF*-J23106_*gfp* | This study | |
| pCDF-Dynamic-*gfp* | pCDF-*tetR*-*phlF*-P*_phlF_*_*infA*-J23106_*gfp* | This study | |
| pCAD | Plasmid containing *cad* gene | (Ye et al, 2022)[41] | |
| pCDF-Dynamic-*cad* | pCDF-*tetR*-*phlF*-P*_phlF_*_*infA*-J23106_*cad* | This study | |
| pCDF-*cad* | pCDF-*tetR*-*phlF*-J23106_*cad* | This study | |

Table S2. Primers used in this study

| Name | Sequence (5′-3′) |
| --- | --- |
| C-infA-F | gtggcgagtccatgttcagccg |
| C-infA-B | aaacctcatgggtggcaacggg |
| D-infA-F | tgccgaataatttctgggtaccacgatgcttgttttcaccacaagaatgagcatgaccggcgcgatgc |
| D-infA-B | ctcgttctttctcttcgcccatcaggcggtaaaacaatcagcgactacgggctcagcggatctcatgcgc |
| TetR-HpaI-R | aggcgaaaatcctgtttgatggtggttaacgacgtcttaagacccactttc |
| phlF-TetR-R | tctcgatgctccttttgggttagtcggatccgatcttttgaattcttttctctatc |
| phlF-TetR-F | ggatccgactaacccaaaaggagcatcgagaatggcacgtaccccgagc |
| phlF-R1 | ggttttttgcggcatacgactggtcgtaagaccgctttaacgctgtgtacccggac |
| phlF-R2 | aagttaagtgatttcacacctacaagagaagcgaaaaaaccccgccgaagcggggttttttgcggcatacgac |
| opt-gfp-R | gtgaccgtgtgcttctcaaatgcctgaggtttcagcaaaaaac |
| opt-gfp-F | gattcgttaccaattgacatgatacgaaacgtaccgtatcgttaaggtattgcgtgagaaaggagc |
| P*_phlF_*-phlF-F | ttctcttgtaggtgtgaaatcacttaacttgattcgttaccaattgacatgatac |
| InfA-P*_phlF_*-F | gattcgttaccaattgacatgatacgaaacgtaccgtatcgttaaggtgtagtactggaaatgagcatcc |
| InfA-PstI-R | ataacagtttactctgcaggtgtgggcccctcagcgactacggaagac |
| lambdaterm-F | ggggcccacacctgcagagtaaactgttatgcttggactcctgttgatag |
| modified-SmR-F | gttttttgcgcctcaggcatttgagaagcacacgg |
| modified-phlF-R | ataatggtaccaaaaaaaaccccgccctgtcaggggcggggtttttttttgcggcatacgactggtcg |
| modified-gfp-F | aatttggtacctttcagcaaaaaacccctc |
| modified-gfp-R | aaatgcctgaggcgcaaaaaaccccgcttc |
| Prefix-hom-R | ttctcttgtaggtgtgaaatcacttaacttgaattcgcggccgcttctaga |
| BBaB1002-hom-F | aagttaagtgatttcacacctacaagagaagcgaaaaaaccccgccgaag |
| cad-AvrII-R | aaaaacctaggttaattaagctgttaatggtg |
| CAD-J23106-F | aaaaacctaggtatagtgctagcaaaaaaaacaaaaggagcatcac |
| D-infA-F2 | tgccgaataatttctgggtaccacgatgcttgttttcaccacaagaatagtgctggagcgaactgc |
| D-infA-B2 | ctcgttctttctcttcgcccatcaggcggtaaaacaatcagcgactacggggagtactcgcggttgactg |
| qPCR_cloDF13_F | tgttcacttgagtccaaccc |
| qPCR_cloDF13_R | ttctgcgcgtaatcttttgc |
| qPCR_rpoA_eco_F | ggcggtgagagttcagggc |
| qPCR_rpoA_eco_R | cgttctcatcggtcaggtggc |
| Flank_rpoA_eco_F | cgtttaagcgtaagccggagcg |
| Flank_rpoA_eco_R | gctcaactacgcggcgcagc |
| RT-infA-F  RT-infA-R  cysG-RT-F  cysG-RT-R | accatgttccgcgtagagtt  gcgactacggaagacaatgc  ttgtcggcggtggtgatgtc  atgcggtgaactgtggaataaacg |

**Supplementary Methods.**

**Measurement of relative expression levels of *infA***

The relative expression of *infA* per plasmid was assessed by real-time quantitative reverse transcription PCR (qRT-PCR). After 10 hours of aTc (0.5, 5, 50 ng/mL) addition to DCG, cell cultures were sampled and combined with twice their volume of RNAprotect Bacteria Reagent. (Qiagen, Hilden, Germany). RNA extraction was conducted using RNeasy Plus Mini Kit (Qiagen). RNase-free DNase set (Qiagen) was used for on-column DNase treatment. A total of 180 ng of extracted RNA was utilized for qRT-PCR with the Luna® Universal One-Step RT-qPCR kit (New England Biolabs, Massachusetts, USA). The *cysG* gene (cysG-RT-F, cysG-RT-R) was used as the reference gene for the quantification of *infA* expression (RT-infA-F, RT-infA-R). The relative ~~amount of~~ *infA* expression level of a cell was calculated by $2^{-\Delta\Delta Ct}$. The relative amount of *infA* of a plasmid was obtained following the formula below.

$$The relative amount of infA m\mathrm{RNA} per plasmid=\frac{The relative amount of infA per cell}{\mathrm{PCN}}$$

**Evaluation of expression levels of CAD**

The expression of CAD protein was analyzed using SDS-PAGE. Cell culture after 24 hours of aTc induction was centrifuged and lysed with lysis buffer (100 mM NaH2PO4, 10 mM Tris-HCl) and 0.5 mg/mL lysozyme. For the total fraction, 5M urea was additionally added. After 30 minutes of ice incubation, the samples were sonicated using a Q800R Sonicator (Qsonica, Connecticut, USA). OD adjusted total fraction samples and the soluble fraction samples were concentrated 5-fold and adjusted by protein concentration. Samples were loaded to SurePAGE™ 4–12% Bis-Tris gels (GenScript, NJ, USA) with MOPS SDS Running Buffer (Thermo Scientific™, Massachusetts, USA).  Spectra™ Multicolor Broad Range Protein Ladder (Thermo Scientific™) was used for the detection of the target protein. The gel was stained with SimplyBlue™ SafeStain (Invitrogen, California, USA) and destained with DDW.
